# The impact of early thromboelastography directed therapy in trauma resuscitation

**DOI:** 10.1186/s13049-017-0443-4

**Published:** 2017-10-05

**Authors:** Mohamed Mohamed, Karl Majeske, Gul R. Sachwani, Kristin Kennedy, Mina Salib, Michael McCann

**Affiliations:** 1 School of Business Administration, Oakland University, Rochester, MI 48309-4493 USA; 2 Hurley Medical Center, One Hurley Plaza, Flint, MI 48503 USA

**Keywords:** Thrombelastography, Coagulopathy of trauma, Trauma / critical care

## Abstract

**Background:**

Conventional coagulation tests do not provide an accurate representation of the complex nature of trauma induced coagulopathy. Thrombelastography provides a prompt global overview of all dynamic sequential aspects of trauma induced coagulopathy. The objective of this study was to evaluate the impact of using thrombelastography on blood products utilization, crystalloids utilization, hospital, and intensive care using length of stay, and cost savings.

**Methods:**

We retrospectively reviewed 134 patients (May of 2012 to February of 2015) meeting Class I trauma activation. Outcome data was compared between two groups: patients prior to thrombelastography implementation (*n* = 87) and patients with thrombelastography guided trauma resuscitation (*n* = 47). Blood product usage was compared for three time periods: first 4 h, the next 20 h, and first 24 h.

**Results:**

For the first 24 h of treatment, patients with thrombelastography guided trauma resuscitation had lower packed red blood cells (*p* = 0.0022) and fresh frozen plasma (*p* = 0.0474), but higher jumbo pack platelets (*p* = 0.0476) utilization when compared to the patients prior to thrombelastography implementation. There was no statistical significant difference in the utilization of crystalloids for any of the three time intervals. Patients with thrombelastography guided trauma resuscitation had a shorter hospital length of stay (*p* = 0.0011) and intensive care unit length of stay (*p* = 0.0059) than the patients prior to thrombelastography implementation. Cost savings in blood products transfusion were most pronounced in patients with penetrating injuries.

**Discussion:**

Using visco-elastic tests to guide blood transfusion was first used for liver transplant patients and then applied to cardiovascular surgery and trauma. Similar to other studies, this study showed using visco-elastic tests for trauma patietns corresponded to an overall reduction in the use of packed red blood cells and fresh frozen plasma during the first 24 hours of resuscitation. In addition, this study showed using visco-elastic tests corresponded to a significant reduction in both hospital and intensive care unit length of stay.

**Conclusion:**

This study demonstrates that Thrombelastography guided trauma resuscitation decreases the overall transfusion requirements of packed red blood cells and fresh frozen plasma. However, given the nature of under-recognized jumbo pack platelets dysfunction in the conventional laboratory parameters, jumbo pack platelets utilization is higher when following Thrombelastography directed resuscitation. The utilization of Thrombelastography corresponded to a reduction in hospital length of stay, intensive care unit length of stay and cost of transfused blood products.

**Electronic supplementary material:**

The online version of this article (10.1186/s13049-017-0443-4) contains supplementary material, which is available to authorized users.

## Background

Severe blood loss leading to hemorrhagic shock represents a major factor in trauma related deaths. An intrinsic dysregulation of the coagulation cascade known as trauma induced coagulopathy (TIC) [1] – contributes to the ongoing hemorrhage. One in every four trauma patients presents to the hospital with established TIC that may lead to a sequence of deleterious events [2–4]. Effects of TIC include uncontrolled bleeding, massive transfusion, and multi-organ failure. Further, TIC corresponds to having longer intensive care unit (ICU) and hospital length of stay (LOS), as well as a fourfold increase in mortality [5]. Conventional coagulation tests (CCTs) offer valuable information in monitoring the different aspects of coagulation. However, CCTs have several limitations: (1) they fail to delineate the complex nature of TIC, (2) they are time consuming, and (3) they have questionable value in guiding transfusion requirements [6, 7]. A systematic review in 2011 suggested that CCTs are insufficient to diagnose TIC [8, 9]. Thrombelastography (TEG - Heamonetics Corp, Braintree, MA USA) or rotational thromboelastometry (ROTEM - TEM international; GmbH, Munich, Germany) provides an alternative diagnostic modality for TIC that overcomes the aforementioned limitations. And ROTEM are visco-elastic tests offering a prompt global overview of all dynamic sequential aspects of the TIC process by providing data on the speed of coagulation initiation, kinetics of clot growth, clot strength, and its breakdown [6, 10]. Clinicians have developed massive transfusion protocols (MTP) to prioritize the correction of coagulopathy by delivering blood products in a systematic and coordinated fashion. Moreover, by using visco-elastic tests, physicians can obtain a more detailed assessment of all stages of hemostasis that allows developing a patient specific goal directed therapy [6, 11].

In trauma patients with TIC, evidence exists that visco-elastic tests supersede CCTs in delineating the process of the clotting cascade and therefore are useful in guiding blood products transfusion [7]. Recent guidelines on the management of TIC encourages a treatment strategy focused on goal directed resuscitation with more restriction in the use of intravenous fluids during initial resuscitation [10]. Indiscriminate administration of crystalloids, albumin, or even packed red blood cells (PRBCs) may cause cardiac and pulmonary complications, gastrointestinal dysmotility, and coagulation disturbances. Hence, visco-elastic tests can provide detailed information on which blood products would prove most efficacious to the hemorrhaging trauma patient and accordingly help guide transfusion therapy in appropriate quantities [5, 12]. Since the bleeding trauma patient often requires a significant amount of resources, goal directed restriction in blood products may also help with reducing the economic burden of caring for these patients [10].

There is a paucity in the literature on studies that attempt to evaluate relationships between the use of visco-elastic tests and resuscitation outcomes and costs in trauma patients [13]. The aim of this study was to evaluate the impact of using TEG on 4 main outcomes: (1) Blood products utilization; (2) crystalloids utilization; (3) hospital LOS and ICU LOS; and (4) the cost of utilized blood products and length of stay.

## Methods

### Patients

**Fig. 1:**
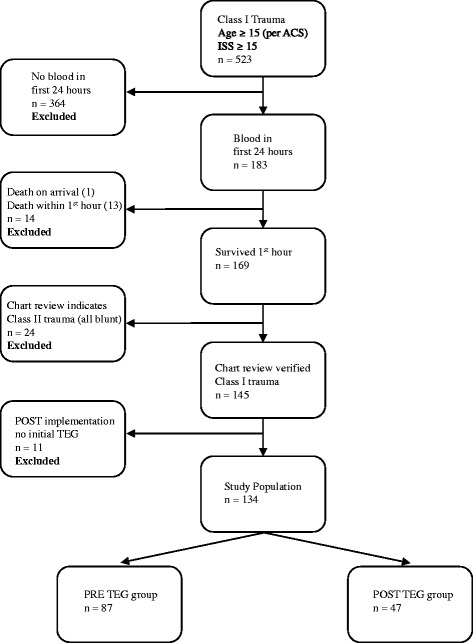
Study population with exclusion criteria

Prior to inception, this study was reviewed and approved by the Institutional Review Board (IRB) at Hurley Medical Center, a university affiliated Level I trauma center. The study consisted of a retrospective analysis on the 4 main outcomes associated with implementation of TEG (a visco-elastic test) for Class I trauma patients (defined as satisfying any of the criteria in Table 1). The primary outcomes were volume of blood products transfused (PRBCs), fresh frozen plasma (FFP), jumbo pack platelets (PLs) (1 jumbo pack = 6 units of platelets), and cryoprecipitate (CRYO) pools (1 pool = 5 units). Secondary outcomes included volume of crystalloids transfused, mortality rates, hospital & ICU LOS, and associated costs. We compared outcomes among two 16 month cohorts of patients: patients prior to TEG implementation (preTEG) - from May 1, 2012 to September 30, 2013; and patients with TEG guided trauma resuscitation (postTEG) - from October 1, 2013 to Feb 28, 2015. To identify subjects for the study we queried the trauma registry for Class I trauma patients between May 1, 2012 and Feb. 28, 2015. We initially idendified 523 patients who were 15 years or older with an injury severity score (ISS) of ≥15 (see Fig. 1). We then excluded 364 patients who did not receive transfusion of blood products within the first 24 h of presentation. Additional exclusions were 13 patients who had a hospital LOS of less than 0.05 days (~1 h) and 1 patient who was pronounced dead upon arrival. Additionally, we excluded 11 patients from the postTEG group who did not receive the initial TEG (reasons included lab error, not following the protocol and the TEG order overridden by the treating surgeon.)

**Table 1:** Criteria for Class I trauma activation

·Glasgow Coma Scale ≤8 with mechanism attributed to trauma
·Systolic blood pressure < 90 at any time in the prehospital or hospital setting
·Respiratory rate < 10 or >29 with respiratory compromise and/or inhalation injury in need of emergent intubation
·Any intubated patient with mechanism attributed to trauma
·Gun-shot wounds to head, neck, chest, abdomen, back or buttocks
·Penetrating injuries to extremities proximal to the elbow or knee
·Stab wounds to the head, neck, cardiac box, torso, or back
·Transfer in from outside hospital receiving/received blood to maintain vital signs
·All transfers in from outside hospital (meeting class I criteria
·Electric burn with cardiac dysrhythmia or significant tissue damage
·Emergency physicians discretion when full trauma team is needed

For the 134 patients meeting the inclusion criteria, we collected basic demographic data: age, gender, mechanism of injury, ISS, and administration of tranexamic acid (TXA) during initial resuscitation. We also obtained data on blood products – volume of PRBCs, FFP, PLs, CRYO, and crystalloids, for three time-periods: 0–4 h, 4–24 h, and 0–24 h. In addition, we recorded ICU and hospital LOS. Data on cost of blood product units was obtained from our blood bank for PRBCs ($193/unit), FFP ($56/unit), PLs ($540/jumbo unit), and CRYO ($259/unit). ICU and hospital charges were obtained from the Finance and Billing department.

### Rapid TEG (r-TEG) assay

Prior to the TEG implementation date (October 1, 2013) staff trauma surgeons were educated on identification of TEG tracings and interpretation of TEG values. During the primary evaluation of patients (usually within 5 min of emergency department admission), a rapid thrombelastography (r-TEG) assay was obtained as part of the initial trauma work-up panel (see Additional file 1 for TEG guidelines). The r-TEG consists of using Kaolin and Tissue factor as reagents to decrease clot initiation time, thus providing results within minutes. The process entails sending a sample of blood to the emergency department lab for analysis on the TEG analyzer. The sample is placed in a cup and heated to body temperature, then rotated in a constant, arc like manner to imitate the venous circulation and stimulate the coagulation process. Following the insertion of a fixed pin attached to a torsion wire into the cup, fibrin begins to form between the wall of the cup and the torsion wire. The strength and speed of clot formation is then transduced into electrical signals that are analyzed by a computer.

The r-TEG assay includes four key values: the R-value (representing time to clot initiation), the K-value (that quantifies fibrin kinetics or speed of clot formation), the α-angle (indicating cross-linking of fibrin), and the maximum amplitude value (MA) (indicating clot strength). The r-TEG test results and tracings were made available for interpretation on a computer in the trauma bay within 5 min of the laboratory receiving the blood sample. Specific replacement algorithms on the basis of TEG interpretations have been described in previous studies [14]. Based on the literature, the following TEG values were used for TEG directed resuscitation: activated clotting time (ACT) > 128, r-value >1.1, k-time > 2.5, α-angle <56, MA < 55, and fibrinolysis at 30 min (LY30) > 3%. Ordering of additional TEG testing, beyond the initial test, was based on physician judgement and discretion.

### Statistical analysis

For quantitative variables, two-sample t-test and the Wilcoxon-Ranksum tests were used for comparing groups of patients on continuous variables with Fisher’s Exact test used for comparing binary variables. Multiple regression was used to perform a multivariate analysis of hospital and ICU LOS and multivariate logistic regression was used to develop a model of patient mortality. JMP Pro version 12.0.1 was used for conducting the analysis. Statistical tests were deemed significant for *p*-values less than 0.05.

## Results

Table 2 contains basic patient demographics for the 134 patients (87 in the preTEG group and 47 in the postTEG group) who met the inclusion criteria. No significant differences were appreciated between the preTEG and postTEG groups. A higher rate of TXA administration was noted in the postTEG group (5.8% preTEG and 14.9% postTEG) yet the difference was not statistically significant (*p* = 0.0757.)

**Table 2:** Basic patient characteristics

	All Patients (*n* = 134)	PRE TEG (*n* = 87)	POST TEG (*n* = 47)	*p*-value
***Age in Years (SD)*** ^***a***^	40.92 ± (19.4)	39.34	43.85	0.1004*
Gender				
Male	101 (75.4%)	65 (74.7%)	36 (76.6%)	0.4918
Female	33 (24.6%)	22 (25.3%)	11 (23.4%)	
Mechanism of Injury				
Blunt	85 (63.4%)	54 (62.1%)	31 (66.0%)	0.4001
Penetrating	49 (36.6%)	33 (38.0%)	16 (34.0%)	
***ISS (mean)***	28.76	28.85	28.60	0.4540
TXA				
Yes	12 (9.0%)	5 (5.8%)	7 (14.9%)	0.0757
No	122 (91.0%)	82 (94.3%)	40 (85.1%)	

^a^ *Standard deviation*

* p-value < 0.05

### Transfused blood products and crystalloids

Table 3 illustrates the differences in blood products and crystalloids administration by time and mechanism of injury.

**Table 3:** Blood products utilization in the study population and stratified by injury mechanism

	All Patients (*n* = 134)				Blunt Injuries (*n* = 85)				Penetrating Injuries (*n* = 49)			
	PRE TEG (*n* = 87) *(units/ patient)*	POST TEG (*n* = 47) *(units /patient)*	Change *(units/ patient)*	*p*-value	PRE TEG (*n* = 54) *(units/ patient)*	POST TEG (*n* = 31) *(units/ patient)*	Change *(units/ patient)*	*p*-value	PRE TEG (*n* = 33) *(units/ patient)*	POST TEG (*n* = 16) *(units/ patient)*	Change *(units/ patient)*	*p*-value
First 4 h												
*PRBCS*	2.48	3.60	1.11	0.0872	2.02	3.16	1.14	0.1072	3.24	4.44	1.20	0.2243
*FFPs*	1.84	3.72	1.88	0.0149*	1.31	3.29	1.98	0.0250*	2.70	4.56	1.87	0.1229
*Platelets*	0.26	1.94	1.67	0.0258*	0.19	0.81	0.62	0.0442*	0.39	4.13	3.73	0.0633
*Cryo*	0.26	0.23	−0.03	0.4297	0.22	0.13	−0.09	0.3420	0.33	0.44	0.10	0.3432
*Crystalloids (L)*	1.62	2.13	0.51	0.0990	2.01	1.62	−0.39	0.1983	0.98	3.11	2.12	0.0037*
Next 20 h												
*PRBCS*	5.21	0.49	−4.72	< 0.0001*	3.22	0.55	−2.67	0.0007*	8.45	0.38	−8.08	0.0003*
*FFPs*	4.59	0.57	−4.01	< 0.0001*	2.59	0.58	−2.01	0.0027*	7.85	0.56	−7.29	0.0001*
*Platelets*	0.56	0.34	−0.22	0.1523	0.37	0.52	0.15	0.3083	0.88	0.00	−0.88	0.0011*
*Cryo*	0.21	0.15	−0.06	0.3573	0.17	0.06	−0.10	0.2241	0.27	0.31	0.04	0.4588
*Crystalloids (L)*	5.38	5.36	−0.02	0.4879	5.68	4.31	−1.36	0.0553	4.91	7.39	2.48	0.0709
24 h												
*PRBCS*	7.69	4.09	−3.60	0.0022*	5.24	3.71	−1.53	0.1079	11.70	4.81	−6.88	0.0004*
*FFPs*	6.43	4.30	−2.13	0.0474*	3.91	3.81	−0.10	0.4884	10.55	5.13	−5.42	0.0174*
*Platelets*	0.83	2.28	1.45	0.0476*	0.56	1.32	0.77	0.0474*	1.27	4.13	2.85	0.1188
*Cryo*	0.47	0.38	−0.09	0.3514	0.39	0.19	−0.20	0.2281	0.61	0.75	0.14	0.3748
*Crystalloids (L)*	7.00	7.49	0.48	0.3317	7.68	5.93	−1.75	0.0649	5.89	10.50	4.60	0.0273*

* p-value < 0.05

#### The first 4 h

Overall, TEG implementation corresponded to an increase in the use of FFP and PLs. Specifically, the postTEG group received an additional 1.67 units of FFP (*p* = 0.0258) and 1.88 units of PLs (*p* = 0.0149) per patient when compared to the preTEG group. In patients with blunt injuries, usage of FFP and PLs were similarly higher in the postTEG group (1.98 units/patient, *p* = 0.0250; 0.62 units/patient, *p* = 0.0442). However, there were no significant differences in the utilization of blood products for patients with penetrating injuries. Patients with penetrating injuries (*n* = 49) demonstrated a significant increase in the volume of transfused crystalloids (2.12 L/patient; *p* = 0.0037), while no difference in crystalloids was observed in patients with blunt injuries.

#### Next 20 h

For all study patients, we found an overall reduction in the use of PRBCs and FFP (4.72 units/patient, *p* = <0.0001 and 4.01 units/patient, *p* = <.0001, respectively) in the next 20 h. While these reductions in PRBCs and FFP were observed for both mechanisms of injury, reductions were more remarkable in patients with penetrating injuries (8.08 units/patient, *p* = 0.0003 and 7.29 units/patient, *p* = 0.0010) than those with blunt injuries (2.67 units/patient, *p* = 0.0007, and 2.01 units/patient, *p* = 0.0027). Additionally, patients with penetrating injuries demonstrated a significant reduction in PLs usage (0.88 units/patient, *p* = 0.0011).

#### Overall 24 h

Overall, significant reductions were observed in the utilization of PRBCs and FFP (3.60 units/patient, *p* = 0.022 and 2.13 units/patient, *p* = 0.0474 respectively) in the first 24 h, however, there was an increase in PLs utilization (1.45 units/patient, *p* = 0.0476). Based on mechanism of injury, penetrating injury patients had significant reductions in PRBCs and FFP (6.88 units/patient, *p* = 0.0040 and 5.42 units/patient, *p* = 0.0174) with significantly higher transfusion volumes of crystalloids (4.60 L/patient, *p* = 0.0273). On the other hand, patients with blunt injuries had significantly higher transfusion volumes of PLs (0.77 units/patient, *p* = 0.0474).

#### Penetrating injuries

Patients with penetrating injuries (*n* = 49) demonstrated a significant increase in the volume of transfused crystalloids (2.12 L/patient; *p* = 0.0037), while no difference in crystalloids was observed in patients with blunt injuries. To better understand this difference, we performed a sub-group analysis. Thirty six patients (73.5%) with penetrating injuries underwent operative intervention versus 13 who were managed non-operatively (see Additional file 2). We compared crystalloids usage among the latter two subgroups and found that operative patients received more crystalloids at all time intervals than non-operative patients (1st 4 h = 1.16 L/patient, *p* = 0.0082; next 20 h = 4.00 L/patient, *p* = 0.0002; 24 h = 5.17 L/patient, *p* = 0.0003).

#### Operative intervention

Of the study population, 67 patients were found to have underwent operative intervention (31 blunt and 36 penetrating). In order to understand the impact of TEG on these 67 patients, we conducted a subgroup analysis to compare the 45 preTEG and 22 postTEG operative patients for utilization of blood products and crystalloids (see Additional file 3). Operative patients in postTEG groups demonstrated the following: during the first 4 h there were significant increases in the utilization of PRBCs, FFP, PLs and crystalloids (*p* = 0.0452, 0.0461, and 0.0055 respectively), while over the next 20 h, significant reductions in PRBCs, FFP and PLs utilization were observed (*p* = <0.0001, <0.0001, and 0.0005 respectively). For the overall 24 h study period, significant reductions in the utilization of PRBCs and FFP were observed (*p* = 0.0035, 0.0140 respectively).

#### Repeat TEGs

The TEG protocol only specified an initial TEG, yet we found that within the first 24 h, 22 of the 47 patients (46.8%) in the postTEG group received a repeat TEG either once (15 patients) or twice (7 patients). Since obtaining repeat TEGs was not in the protocol, we compared the volume of blood products and crystalloids transfused based on the number of TEGs obtained. Specifically, we compared the 25 patients who only had the initial TEG to the 22 patients who had repeat TEGs (see Additional file 4). No statistically significant differences existed in age, ISS, and mechanism of injury. Mortality rate was higher in the initial TEG vs the repeat TEG group (44.0% vs 22.7% respectively), however, the difference was not statistically significant (*p* = 0.1095). TXA administration was also higher in the initial TEG vs the repeat group (92.0% vs 77.3% respectively), however, the difference was also not statistically significant (*p* = 0.1580). The repeat TEG group had higher transfusion rates of all 4 blood products and crystalloids. A majority of the additional PRBCs, FFP and PLs were transfused during the first 4 h, while transfusion of crystalloids was higher in the next 20 h.

### Length of stay

The average hospital LOS for all 134 patients was 17.8 days with no significant difference (*p* = 0.4463) between blunt injury patients (18.0 days) and penetrating injury patients (17.4 days). Overall, patients in the postTEG group were found to have a mean hospital LOS 10.0 days shorter than the preTEG group (11.3 days versus 21.3, *p* = 0.0011) with a reduction of 10.3 days for blunt injury patients (11.4 days vs 21.7 days, *p* = 0.0047), and a reduction of 9.3 days for penetrating injury patients (11.2 days vs 20.5 days, *p* = 0.0513). Table 4 contains the coefficients for a multiple regression model that uses hospital LOS as the dependent variable. This model suggests the older the patient the longer the hospital LOS; the higher the ISS the longer the hospital LOS; and that patients who did not survive had a shorter hospital LOS. The model further suggests that once corrected for age, ISS and mortality, the patients in the postTEG group had a hospital LOS 11.37 days shorter on the average than patients in the preTEG group.

**Table 4:** Multi-Regression model coefficients for Hospital and ICU LOS

	Hospital LOS		ICU LOS	
	*n* = 134		*n* = 119	
Term	Estimate	*p*-value	Estimate	*p*-value
Intercept	14.45	0.0100	5.54	0.2714
Post TEG	−11.37	0.0011	−8.35	0.0093
Age	0.19	0.0301	0.16	0.0482
ISS	0.31	0.0281	0.35	0.0061
Death	−25.34	<.0001	−17.42	<.0001

The average ICU LOS for the 119 patients admitted to the ICU was 14.0 days with no significant difference (*p* = 0.3235) between the 77 blunt injury patients (14.6 days) and the 42 penetrating injury patients (13.0 days). Overall, patients in the postTEG group were found to have an ICU length of stay 7.0 days shorter than patients in the preTEG group (9.3 days versus 16.3 days, *p* = 0.0059). Based on mechanism of injury, blunt injury patients in the postTEG group had a shorter ICU LOS of 6 days (10.6 days versus 16.6 days, *p* = 0.0390), while in penetrating injury patients, ICU LOS was reduced by 9.1 days (6.7 days versus 15.8 days, *p* = 0.0299). Table 4 contains the coefficients for a multiple regression model that uses ICU LOS as the dependent variable. This model suggests the older the patient the longer the ICU LOS; the higher the ISS the longer the ICU LOS; and that patients who did not survive had a shorter ICU LOS. The model further suggests that once corrected for age, ISS and mortality, the patients in the postTEG group had an ICU LOS 8.35 days shorter on the average than patients in the preTEG group.

### Mortality

The overall mortality rate for the 134 patients was 35.82% with no significant difference observed between the preTEG and postTEG groups (36.78% versus 34.04%; *p* = 0.7519). Further, no difference was observed when patients were compared by mechanism of injury (Blunt 37.65% versus Penetrating 32.65%; *p* = 0.5602); administration of tranexamic acid (Yes 50.0% versus No 34.43%; *p* = 0.2921); and operative intervention (Yes 32.84% versus No 38.81%; *p* = 0.4709). As expected, mortality rate was significantly higher in patients with ISS > 25 (Yes 48.48% versus No 23.53%; *p* = 0.0024). To evaluate the effect of age, we grouped the patients into two groups: <30 years (*n* = 54) and ≥30 years (*n* = 80). The mortality rate was significantly lower in patients <30 years in the postTEG group (preTEG 42.5% versus postTEG 14.29%; *p* = 0.0451), while no significant difference was observed in patients that were ≥30 years (preTEG 31.91% versus postTEG 42.42%; *p* = 0.3368).

To assess the overall impact of TEG implementation, we modeled mortality using multivariate logistic regression. The model included age, gender, mechanism of injury, location(s) of injury (based on the six abbreviated injury severity (AIS) score parameters: head/neck, chest, extremity, face, abdomen and external), ISS > 25, administration of tranexamic acid, total blood products transfused in the first 4 h, operative intervention, and ICU admission. The odds ratio for the postTEG:preTEG group was 0.33 (*p* = 0.0334, 95% CI 0.11–0.92). This suggests that once adjusted for the above variables, patients after TEG implementation had one third the mortality rate than prior to TEG implementation.

### Cost of blood products utilization

Overall during the first 24 h, there was a $695 and $119 – $814 in total – cost savings per patient due to the decrease in utilization of PRBCs (3.60 units x $193) and FFP (2.13 units x $56) respectively in the postTEG group when compared to the preTEG group. However, the overall increase in PLs utilization resulted in an increase in costs of $783 per patient (1.45 units x $540). Based on mechanism of injury, reduction in costs were more pronounced in patients with penetrating injuries in whom $1328 and $304 – $1632 in total – in cost savings were incurred for the reduction in utilization of PRBCs (6.88 units x $193) and FFP (5.42 units x $56) respectively. To the contrary, patients with blunt injuries incurred an additional cost of $416/patient for the increase in PLs utilization (0.77 units x $540).

## Discussion

TEG is a dynamic, visco-elastic test that monitors the thrombodynamic properties of blood as a clot is induced. Prior studies have established that visco-elastic tests are superior to CCTs in recognizing TIC resulting in earlier and more accurate intervention [11]. By providing a more comprehensive description of global hemostatic functional aspects, visco-elastic tests produce tracings that correspond to coagulation disturbances allowing a more ‘targeted’ blood component replacement approach. By directing blood transfusion therapy in trauma patients, visco-elastic tests may serve as a useful and cost-effective tool in patients with TIC or those requiring massive blood transfusion. In addition, using visco-elastic tests can potentially help avoid complications associated with MTP. Holocomb et al. demonstrated that using visco-elastic tests provide results faster and cheaper than CCTs, and are strongly associated with clinical outcomes of interest for severely injured trauma patients [15].

The notion of using visco-elastic tests to ‘guide’ blood transfusion was derived after the study by Kang et al. where a TEG-based transfusion algorithm was used during treatment of liver transplant patients [16]. In that study, the TEG based approach resulted in less blood products usage than transfusion guided by the CCTs [16]. Thereafter, interests developed for using visco-elastic test guided transfusion algorithms in cardiovascular surgery and trauma [17]. Tapia et al. demonstrated that TEG-guided resuscitation was superior to standard MTP in patients with penetrating injury that received more than 10 units of blood [14]. The study by Tauber et al. further adds to the evidence that rotational thromboelastometry assays are useful in managing trauma patients [18].

Our study results demonstrate that after TEG implementation, despite an initial increase in the use of FFP and PL during the first 4 h of trauma resuscitation, an overall reduction in the use of PRBCs and FFP was observed during the first 24 h similar to other studies [11, 19, 20]. In the study by Gonzales et al. [11], patients randomized to either MTP goal directed by visco-elastic test or by CCTs. The volumes of PRBCs, FFP, PLs, CRYO and crystalloids were compared at 2, 4, 6, and 12 h [11]. Similar to our results, they revealed significantly higher volumes of FFP and platelets in the first 2 h of resuscitation in the visco-elastic test directed MTP group compared to the CCT group [11]. However, contrary to our results, no difference in the use of PRBCs across all times was observed in their study. We explain this difference by our ‘hemostatic resuscitation’ strategy – early blood products administration and less crystalloids during initial resuscitation – adopted around the same time TEG use was implemented at our center. Nonetheless, the effect of TEG implementation during initial resuscitation was evident by the utilization of lower volumes of PRBCs and FFP when assessed over the first 24 h period. Schochl et al. [19] similarly demonstrated a reduction in PRBCs as well as PLs in TEG directed resuscitation with fibrinogen concentrate and prothrombin concentrate complex. More recently, Stein et al. [20] also demonstrated a reduction in PRBCs and FFP during the period after TEG implementation, and noted a significantly higher rate in use of TXA (similar to our study) and coagulation factor XIII during that period. With regards to the higher use of PLs in our study, we experienced difficulty in extracting data pertinent to the history of antiplatelet use prior to trauma from patients’ charts. However, clinically significant platelet dysfunction following trauma has been previously documented [21].

Studies have shown that using red blood cell transfusion by itself is associated with greater risk of infection, increased hospital stay, costs and mortality [7]. Significant evidence exists of increased morbidity and mortality in patients receiving excessive transfusions, resulting in continued efforts to safely reduce the use of blood products in severely injured or hemorrhagic patients [5]. The 2016 European guidelines on management of major bleeding and coagulopathy following trauma encourages restriction in volume replacement during resuscitation and guidance by goal-directed strategies [10]. Several authors advocate the use of visco-elastic tests to guide and reduce blood product requirements during resuscitation by transfusing on the basis of specific component requirements [19, 22–24]. In a recent single-center, parallel-group, open-label, randomized trial, the use of fibrinogen supplementation for severe clotting failure in multiple trauma demonstrated superiority over FFP [25]. This evolving visco-elastic test based strategy does not require adhering to the fixed-ratio approach used in most MTP. Although a fixed-ratio strategy attempts to mimic whole blood concentrations and improves survival, it tends not to occur without complications (described earlier) in those who receive excessive transfusions [5]. Thus, implementation of strategies that both avoid (a) excessive transfusion and (b) provide appropriate and adequate replacement while preserving these ratios, seem sensible. Our study demonstrates that using TEG, generates these types of results. We observed reductions in the volume of PRBCs and FFP utilized in the first 24 h of resuscitation with reductions being more pronounced in patients with penetrating injuries – who often require massive transfusion – while maintaining our PRBCs:FFP ratio at 1:1 (observed from our monthly performance improvement metrics).

Similarly, Tapia et al. demonstrated comparable transfusion ratios before and after implementation of an MTP [14]. In their study, visco-elastic tests were used only during the pre-MTP period and provided appropriate ratios on the basis of laboratory data (visco-elastic tests) rather than predefined ratios [14]. Studies have shown that following a rigid 1:1:1 (PRBCs:FFP:PLs) transfusion protocol, may lead to excessive administration and unnecessary exposure to blood components, and increase the risk of inflammation, acute respiratory distress syndrome (ARDS), sepsis, multiple organ failure, and mortality [10, 26, 27]. Protocols that include early and individualized goal-directed coagulation therapy are rapidly evolving and continue to replace the predefined ratio-driven protocols [6].

In a RCT by Gonzalez et al., no significant difference in the amounts of crystalloids transfused in the first 24 h in conjunction with a visco-elastic test based protocol was observed [11]. To the contrary, our results demonstrate a significant increase in the volume of crystalloids transfused in patients with penetrating injuries during the first 4 and 24 h. We hypothesize this may have resulted from a tendency by our anesthesiologists to inadvertently increase the volume of crystalloids transfused in patients that required operative intervention during the perioperative period. Similar to our results, the study by Tapia et al. revealed that using visco-elastic tests on patients with either blunt or penetrating mechanisms of injury resulted in a significantly higher amount of transfused crystalloids [14].

The overall mortality rates were virtually identical before and after TEG implementation. Even though an adjusted mortality benefit was observed in the postTEG group, the application of our model may not be relevant in the clinical setting.

Upon review of literature, studies that attempted to evaluate the impact of visco-elastic test usage in the trauma setting on hospital and ICU LOS, are scarce. This makes our evaluation of hospital and ICU LOS one of the unique features of this study. Our results demonstrate a significant reduction in both hospital and ICU length of stay in the postTEG group. This was true for both patients with blunt or penetrating injuries. While these reductions are astonishing, it is intuitive to assume that in addition to TEG, several cofounding factors probably contributed to these results. Interestingly, similar to our results, the recent RCT by Gonzalez et al. demonstrated a 7.5 days increase in ICU-free days in the TEG group, however, this result was not statistically significant [11]. When exploring ways to drive down healthcare costs evaluation of length of stay becomes important. In the United States, the ICU represents one of the largest cost drivers in the hospital setting [28].

A systematic review and cost effectiveness analysis by Whiting et al., indicated that viscoelastic testing is both lower in cost and more effective than standard laboratory tests in patients undergoing cardiac surgery and in trauma patients [7]. In 2005, Dasta et al. conducted a study to investigate the daily costs of ICU care and mechanical ventilation across a large and diverse sample of U.S. hospitals [28]. This retrospective analysis of the NDCHealth Hospital Patient Level Database (data from approximately 300 U.S. general medical/surgical hospitals) demonstrated a stable unadjusted daily cost for the first 3 days in the surgical ICU of approximately $3500/day [28]. In trauma patients, the mean daily costs for the first three ICU days were $10,299, $4887, and $3876 for patients requiring mechanical ventilation, and $5973, $3275, and $3059 for patients not requiring mechanical ventilation. Using these figures as a standard we can estimate the range of cost savings associated with the 7 day reduction in ICU length of stay observed in this study. On the low end, by using the $3500/day cost, a 7 day decrease in ICU length of stay would correspond to savings of $24,500. On the high end, a 7 day reduction in ICU length of stay in patients that required mechanical ventilation would correspond to savings of $19,062 for the first 3 days (by adding up the mean costs for days 1–3), and $14,000 for the remaining 4 days (by multiplying $3500 by 4 days), a combined cost savings of $33,062. Besides these ongoing cost savings, it is important to note though that the initial cost incurred by our institution to purchase the TEG analyzer was $53,625. It is estimated that the cost of materials per test is approximately $17.33 [7].

Apart from its retrospective nature, we recognize other limitations to this study. Although we think that reductions in blood products usage would correspond to a decrease in bleeding, we could not assess nor quantify encountered bleeding due to inconsistencies in documentation. Repeat TEGs were performed only on some patients as they were not part of the protocol. This did not enable a more comprehensive analysis on the impact of TEG after initial resuscitation. Due to the small sample size, we could not further explain the increased utilization of blood products in the patients that received repeat TEGs compared to those that received the initial TEG only. Despite the higher mortality rate observed in patients who received the initial TEG only – which may overall represent less volumes of blood products transfusion because of death – relative to patients who received repeat TEGs, the difference was not statistically significant. Another limitation is that we did not collect data on platelet mappings that may have been performed and which may have enabled better assessment of the differences observed in PLs transfusion. Additionally, it is well known that fibrinogen reaches critical low levels early in trauma [29–31]. Measurement of functional fibrinogen was not part of our TEG protocol and was only obtained based on physician discretion. Thus, we did not collect data on fibrinogen levels. We also experienced difficulty exporting data on TEG parameters from the manufacturer’s software for analysis. Although, we did have documentation of a few TEG parameters in electronic medical records, this data was not consistently available for the entirety of the TEG implementation period. Thus, though important, we were unable to report this data. Lastly, our TEG-based transfusion algorithm did not specify the type and amount of transfused blood products based on TEG parameters. Fortunately, several transfusion algorithms that describe both the type and volumes required are recognized and have been well described in previous literature [6]. Trauma surgeons at our institution recognize and utilize these algorithms during practice.

## Conclusion

Our study results demonstrate that using technologies such as TEG to direct trauma resuscitation appears to achieve an overall reduction in utilization of PRBCs and FFP with an increase in the use of PLs in the first 24 h of resuscitation, while maintaining a PRBCs:FFP ratio at 1:1. TEG use was associated with reductions in hospital and ICU length of stay of 10.2 and 7 days for all patients with either blunt or penetrating injury. Estimation of associated costs revealed cost savings of $1632 per patient in blood products utilization during the first 24 h for patients with penetrating mechanism of injury, and an overall cost savings of $24,500 – $33,062 for a 7 day reduction in ICU length of stay.

## Additional file

Additional file 1: Hurley Medical Center TEG guidelines. (DOCX 792 kb)
Additional file 2: Comparison of blood products and crystalloids utilization in patients with penetrating injury based on operative intervention. (DOCX 15.2 kb)
Additional file 3: Comparison of patients with initial vs repeat TEGs. (DOCX 14.8 kb)
Additional file 4: Comparison of the 67 pre and postTEG patients who underwent operative intervention. (DOCX 14.9 kb)

## Abbreviations

(ACT): Activated clotting time
(CCTs): Conventional coagulation tests
(CRYO): Cryoprecipitate
(FFP): Fresh frozen plasma
(ICU): Intensive care unit
(ISS): Injury severity score
(LOS): Length of stay
(LY30): Fibrinolysis at 30 min
(MA): Maximum amplitude value
(MTP): Massive transfusion protocol
(PLs): Jumbo pack platelets
(postTEG): Patients with TEG guided trauma resuscitation
(PRBCs): Packed red blood cells
(preTEG): Patients prior to TEG implementation
(ROTEM): Rotational thromboelastometry
(r-TEG): Rapid thrombelastography
(TEG): Thrombelastography
(TIC): Trauma induced coagulopathy
(TXA): Tranexamic acid
