# Supplementary material for: The impact of early thromboelastography directed therapy in trauma resuscitation
Source: Scand J Trauma Resusc Emerg Med. 2017 Oct 5;25:99. doi: 10.1186/s13049-017-0443-4 (PMC5629752; doi:10.1186/s13049-017-0443-4)
Supplement: Additional file 1: — Hurley Medical Center TEG guidelines. (DOCX 792 kb) [file 13049_2017_443_MOESM1_ESM.docx]

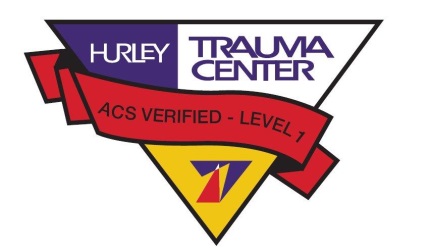
HURLEY MEDICAL CENTER

TRAUMA SERVICES

**TEG GUIDELINES**

r-TEG Guided Resuscitation of Hemorrhage and Coagulopathy in Trauma

Thrombelastography (TEG) is a viscoelastic test whose tracings and values provide a comprehensive illustration of the clotting cascade. TEG values correspond to specific coagulation components and factors that contribute to particular portions of the TEG tracing. Disturbances in the tracings provide diagnostic insight as well as therapeutic purposes. To expedite the delivery of information, some clinicians prefer to use the Rapid TEG (r-TEG) rather than the standard TEG. The addition of tissue factor to kaolin (r-TEG) produces a tracing that yields results 5-10 minutes faster than standard TEG.

The first value that returns in the r-TEG is the activated clotting time (ACT). The ACT represents the time from the start of the assay to the initiation of clot formation. The normal range of ACT in healthy volunteers is 86-118 seconds. The ACT is prolonged in patients with hypocoagulable states related to factor deficiency or severe hemodilution. Patients with significantly prolonged ACT values (>128 seconds) in hemorrhagic shock should receive RBC and plasma-based resuscitation. Patients without frank hemorrhage can be resuscitated with a generous volume of plasma instead.

The r-value or reaction time is similar, in representation terms, to that of the ACT. It too measures the time between the beginning of the assay and the initial formation of clot fibers. Normal r-values range from 0.0 to 1.0 minutes. As with ACT, a prolonged r-value (>1.1 minutes) should be managed with RBC and plasma in those patients presenting with hemorrhage.

The k-time reflects the kinetics of the clot’s formation and is the time in minutes that is needed to reach 20-mm clot strength. The k-time’s normal range is 1–2 minutes and is generally increased with hypofibrinogenemia or platelet deficiency or dysfunction. When a patient’s k-time is >2.5 minutes, one should consider utilizing plasma for resuscitation and correction of coagulopathy. In the presence of an altered alpha angle, cryoprecipitate (or fibrinogen concentrate) should also be transfused.

The alpha angle is the slope of the tracing that represents the rate or acceleration of clot formation. Normal ranges for healthy volunteers are between 66 and 82 degrees. A decreased slop or angle is observed in situations where interactions between factors, fibrinogen and platelets are decreased or disturbed, such as hypofibrinogenemia or platelet deficiency/dysfunction. In situations where the angle is <56 degrees, cryoprecipitate (or fibrinogen concentrates) should be transfused.

The maximal amplitude (mA) is the highest amplitude of the r-TEG tracing and reflects the contribution of platelet count, platelet function and platelet–fibrin interactions to overall clot strength. Normal values in healthy volunteers range from 52-71. Low mA values correspond to situations of platelet dysfunction and or hypofibrinogenemia. In patients with relatively normal alpha angle but mA <55, platelets should be transfused immediately. However, if the angle is abnormal and the mA <55, both platelet and cryoprecipitate (or fibrinogen concentrates) should be transfused.

The LY-30 is the percent amplitude reduction at 30 minutes after achieving the mA and when reflects the balance of clot stability and clot breakdown (fibrinolysis). Normal ranges in healthy volunteers have been reported to occur between 0 and 7.5%. In states of increased fibrinolysis, the LY30% is increased. In injured patients presenting with evidence of hemorrhage or bleeding and an LY-30% >3%, anti-fibrinolytic agents (such as tranexamic acid or amino-caproic acid) should be administered as soon as possible.

**DISCLAIMER: These guidelines were prepared by the Department of Trauma Services, Hurley Medical Center. They are intended to serve as a general statement regarding appropriate patient care practices based upon the available medical literature and clinical expertise at the time of development. They should not be considered to be accepted protocol or policy, nor are intended to replace clinical judgment or dictate care of individual patients.**


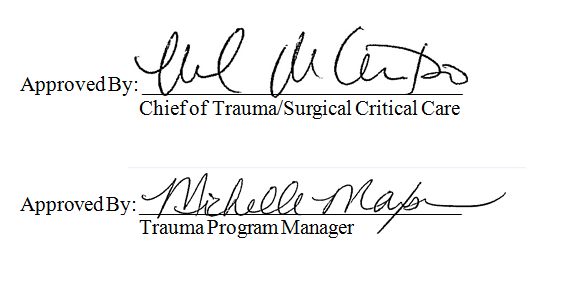


**REFERENCES**

1. Luddington RJ. Thromboelastography/thromboelastometry. *Clin Lab Haematol* 2005;27:81-90.
2. Orlando Regional Medical Center Department Specific Policy #4019. Thromboelastography Analysis. Approved April 2006, last revised March 2013.
3. Wang SC, Shieh JF, Chang KY et al. Thromboelastography-guided transfusion decreases intraoperative blood transfusion during orthotopic liver transplantation: randomized clinical trial. *Transfusion* 2010;47:2590-3.
4. Speiss BD, Gilles BS, Chandler W, et al. Changes in transfusion therapy and re-exploration rate after institution of a blood management program in cardiac surgical patients. *J Cardiothoracic Vasc Anesth* 1995;9:168-173.
5. Ak K, Isbir CS, Tetik S, et al. Thromboelastography-based transfusion algorithm reduced blood product use after elective CABG: a prospective randomized study. *J Card Surg* 2009;24:404-410.
6. Niles SE, McLaughlin DF, Perkins JG et al. Increased mortality associated with the early coagulopathy of trauma in combat casualties. *J Trauma* 2008;64:1459-63.
7. Brohi K, Singh J, Heron M, Coats T. Acute traumatic coagulopathy. *J Trauma* 2003;54:1127-30
8. Cotton BA, Gunter OL, Isbell J et al. Damage control hematology: the impact of a trauma exsanguination protocol on survival and blood product utilization. *J Trauma* 2008;64:1177-82.
9. Cohen MJ, Call M, Nelson M, et al. Critical role of activated protein C in early coagulopathy and later organ failure, infection and death in trauma patients. *Ann Surg* 2012;255:379-385.
10. Cohen MK, Kutcher M, Redick B, et al. Clinical and mechanistic drivers of acute traumatic coagulopathy. *J Trauma Acute Care Surg* 2013;75:S40-47.
11. Kaufmann CR, Dwyer KM, Crews JD et al. Usefulness of thromboelastography in assessment of trauma patient coagulation. *J Trauma* 1997;42:716-20.
12. Martini WZ, Cortez DS, Dubick MA, et al. Thromboelastography is better than PT, aPTT, and activated clotting time in detecting clinically relevant clotting abnormalities after hypothermia, hemorrhagic shock, and resuscitation in pigs. *J Trauma* 2008; 65:535-543.
13. Holcomb JB, Minei KM, Scerbo ML, et al. Admission rapid thromboelastography can replace conventional coagulation tests in the emergency department. *Annals of Surgery* 2012;256:476-486.
14. Cotton BA, Faz G, Hatch QM, et al. Rapid thromboelastography delivers real-time results that predict transfusion within 1 hour of admission. *J Trauma* 2011;71:407-417.
15. Vogel AM, Radwan ZA, Cox CS, et al. Admission rapid thromboelastography delivers real-time “actionable” data in pediatric trauma. *J Ped Surg* 2013; 48:1371-1376.
16. Tapia NM, Chang A, Norman M, et al. TEG-guided resuscitation is superior to standardized MTP resuscitation in massively transfused penetrating trauma patients. *J Trauma Acute Care Surg* 2012; 74:378-386.

Side A of r-­‐TEG card

| r-TEG value  (normal range) | Definition | Interpretation |
| --- | --- | --- |
| ACT  (86-118 seconds) | Time from start of assay to  initiation of clot | Prolonged with factor deficiency or  severe hemodilution |
| r-value  (0.0-1.0 minutes) | Time between beginning of  assay and initial clot formation | Prolonged with factor deficiency or  severe hemodilution |
| k-time  (1.0-2.0 minutes) | Time needed to reach 20-mm  clot strength | Increased with  hypofibrinogenemia or platelet  dysfunction |
| alpha-angle  (66-82 degrees) | Rate or acceleration of clot  formation | Decreased with  hypofibrinogenemia or platelet  dysfunction |
| mA  (54-72 mm) | Contribution of platelet  function and platelet–fibrin  interactions | Decreased with platelet  dysfunction and or  hypofibrinogenemia |
| LY-30%  (0-7.5%) | Amplitude reduction 30  minutes after achieving mA  (degree of fibrinolysis) | Increased with accelerated  fibrinolysis |

Side B of r-­‐TEG card

| ACT> 128 | Transfuse plasma and RBC |
| --- | --- |
| r-value> 1.1 | Transfuse plasma and RBC |
| k-time> 2.5 | Transfuse plasma  Add cryoprecipitate/fibrinogen if angle also abnormal |
| α-angle < 56 | Transfuse cryoprecipitate (or fibrinogen)  Add platelets if mA is also abnormal |
| MA < 55 | Transfuse platelets  Add cryoprecipitate/fibrinogen if angle also abnormal |
| LY-30 > 3% | Administer tranexamic acid or amino-caproic acid |


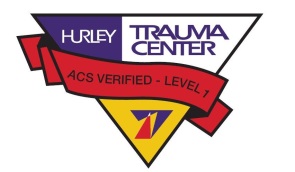

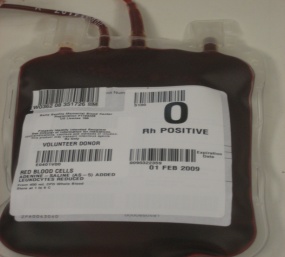
Goal Directed Therapy: Which Products and When?

**_2 u FFP: 800 mg Fibrinogen,_**

**_15cc/Kg=25% increase factors_**


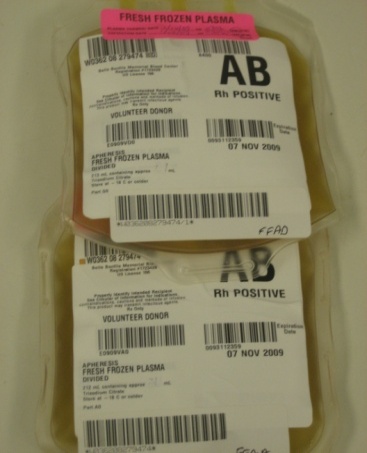


**Coagulation Factors for thrombin generation**

**RBC’s**

**~100 mg Fibrinogen**

**TEG -ACT
>128sec**

**FFP**

**α<56°**

**Cryoprecipitate**


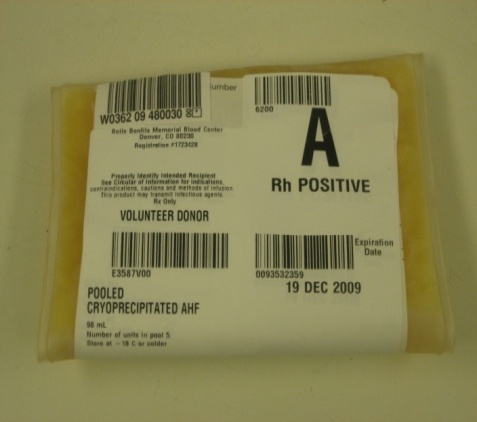


**Thrombin cleaves**

**soluble fibrinogen to fibrin**

**10 u Cryoprecipitate 1500 mg Fibrinogen 800 IU VIII,V,VWF,XIII**

**MA<55mm**

**TEG -ACT
>125sec**


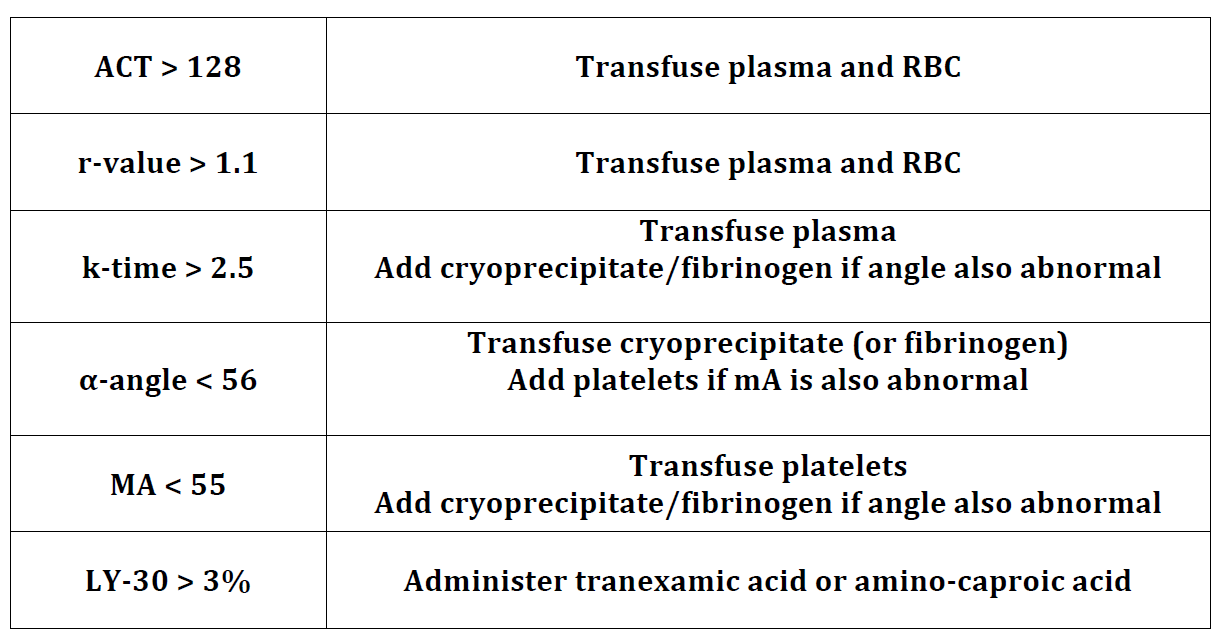

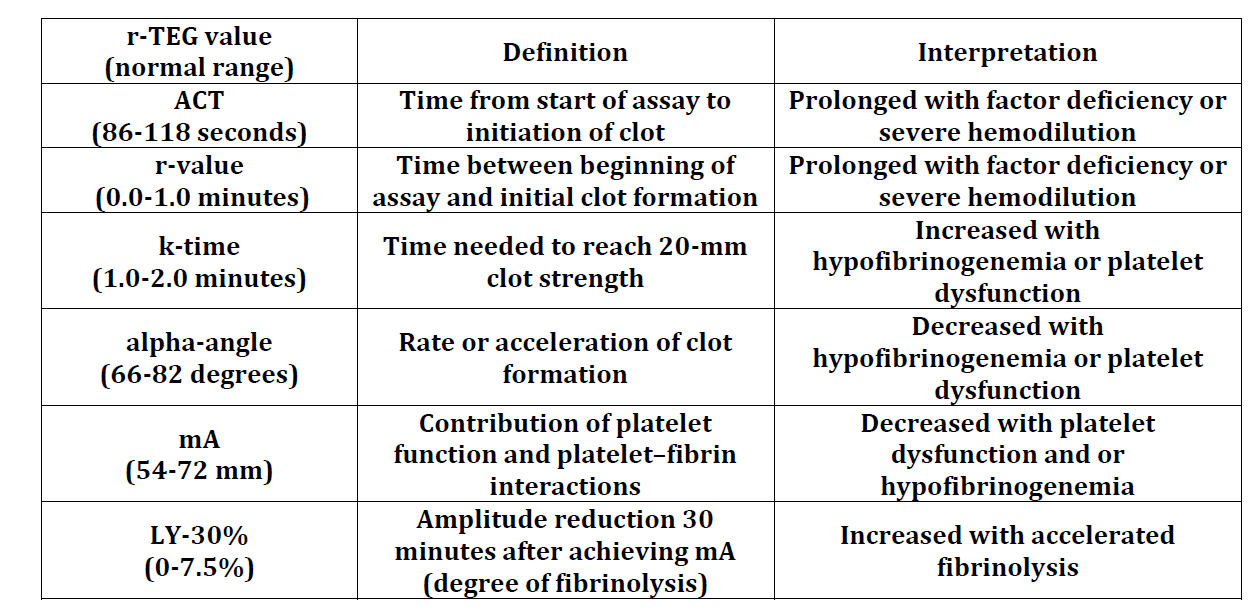


**Apheresis platelets**

**10 u Platelets 300 mg fibrinogen**


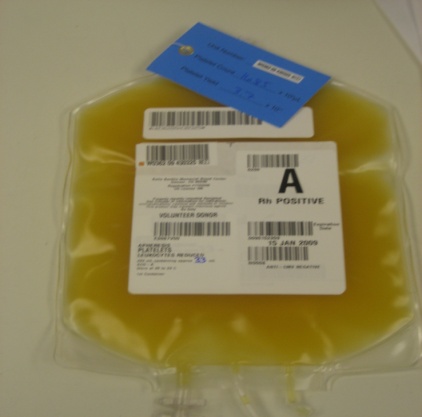

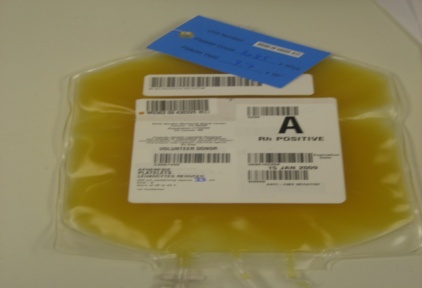


**Thrombin Burst:**

**platelet activation**
