# Supplementary material for: The impact of early thromboelastography directed therapy in trauma resuscitation
Source: Scand J Trauma Resusc Emerg Med. 2017 Oct 5;25:99. doi: 10.1186/s13049-017-0443-4 (PMC5629752; doi:10.1186/s13049-017-0443-4)
Supplement: Additional file 2: — Comparison of blood products and crystalloids utilization in patients with penetrating injury based on operative intervention. (DOCX 15.2 kb) [file 13049_2017_443_MOESM2_ESM.docx]

|  | **Total patients, mean units (n=49)** | **No operative intervention (n=13)** | **Received operative intervention  (n=36)** | **Difference** | **p-value** |
| --- | --- | --- | --- | --- | --- |
| **First 4 hours** |  |  |  |  |  |
| *PRBCS* | 3.63 | 3.23 | 3.78 | 0.55 | 0.3504 |
| *FFPs* | 3.31 | 2.15 | 3.72 | 1.57 | 0.1482 |
| *Platelets* | 1.61 | 1.00 | 1.83 | 0.83 | 0.2500 |
| *Cryo* | 0.37 | 0.31 | 0.39 | 0.08 | 0.3773 |
| *Crystalloids (L)* | 1.68 | 0.82 | 1.98 | **1.16** | **0.0082*** |
|  |  |  |  |  |  |
| **Next 20 hours** |  |  |  |  |  |
| *PRBCS* | 5.82 | 0.23 | 7.83 | 7.60 | **0.0002*** |
| *FFPs* | 5.47 | 0.38 | 7.31 | 6.92 | **0.0007*** |
| *Platelets* | 0.59 | 0.00 | 0.81 | 0.81 | **0.0012*** |
| *Cryo* | 0.29 | 0.15 | 0.33 | 0.18 | 0.2634 |
| *Crystalloids (L)* | 5.72 | 2.78 | 6.78 | **4.00** | **0.0002*** |
|  |  |  |  |  |  |
| **24 hours** |  |  |  |  |  |
| *PRBCS* | 9.45 | 3.46 | 11.61 | 8.15 | **0.0002*** |
| *FFPs* | 8.78 | 2.54 | 11.03 | 8.49 | **0.0002*** |
| *Platelets* | 2.20 | 1.00 | 2.64 | 1.64 | 0.0929 |
| *Cryo* | 0.65 | 0.46 | 0.72 | 0.26 | 0.2458 |
| *Crystalloids (L)* | 7.40 | 3.60 | 8.77 | **5.17** | **0.0003*** |
|  |  |  |  |  |  |

Comparison of blood products and crystalloids utilization in patients with penetrating injury based on operative intervention
