# Supplementary material for: The impact of early thromboelastography directed therapy in trauma resuscitation
Source: Scand J Trauma Resusc Emerg Med. 2017 Oct 5;25:99. doi: 10.1186/s13049-017-0443-4 (PMC5629752; doi:10.1186/s13049-017-0443-4)
Supplement: Additional file 3: — Comparison of patients with initial vs repeat TEGs. (DOCX 14.8 kb) [file 13049_2017_443_MOESM3_ESM.docx]

|  | **Initial TEG only** | **Repeat TEGs** | **Difference** | **p-value** |
| --- | --- | --- | --- | --- |
|  | **(n=25)** | **(n=22)** |  |  |
| **First 4 hours** |  |  |  |  |
| *PRBCS* | 2.76 | 4.55 | 1.79 | 0.0958 |
| *FFPs* | 2.56 | 5.05 | 2.49 | **0.0500*** |
| *Platelets* | 1.28 | 2.68 | 1.40 | 0.2173 |
| *Cryo* | 0.08 | 0.41 | 0.33 | 0.0813 |
| *Crystalloids (L)* | 1.75 | 2.55 | 0.80 | 0.1117 |
|  |  |  |  |  |
| **Next 20 hours** |  |  |  |  |
| *PRBCS* | 0.12 | 0.91 | 0.79 | **0.0119*** |
| *FFPs* | 0.20 | 1.00 | 0.80 | **0.0150*** |
| *Platelets* | 0.24 | 0.45 | 0.21 | 0.2628 |
| *Cryo* | 0.00 | 0.32 | 0.32 | 0.1002 |
| *Crystalloids (L)* | 4.09 | 6.80 | 2.72 | **0.0259*** |
|  |  |  |  |  |
| **24 hours** |  |  |  |  |
| *PRBCS* | 2.88 | 5.45 | 2.57 | **0.0377*** |
| *FFPs* | 2.76 | 6.05 | 3.29 | **0.0147*** |
| *Platelets* | 1.52 | 3.14 | 1.62 | 0.1845 |
| *Cryo* | 0.08 | 0.73 | 0.65 | **0.0249*** |
| *Crystalloids (L)* | 5.84 | 9.36 | 3.52 | **0.0343*** |
|  |  |  |  |  |

Comparison of patients with initial vs repeat TEGs
