# Supplementary material for: The impact of early thromboelastography directed therapy in trauma resuscitation
Source: Scand J Trauma Resusc Emerg Med. 2017 Oct 5;25:99. doi: 10.1186/s13049-017-0443-4 (PMC5629752; doi:10.1186/s13049-017-0443-4)
Supplement: Additional file 4: — Comparison of the 67 pre and postTEG patients who underwent operative intervention. (DOCX 14.9 kb) [file 13049_2017_443_MOESM4_ESM.docx]

|  | **preTEG** | **postTEG** | **Difference** | **p-value** |
| --- | --- | --- | --- | --- |
|  | **(n=45)** | **(n=22)** |  |  |
| **First 4 hours** |  |  |  |  |
| *PRBCS* | 3.11 | 5.55 | 2.43 | 0.0508 |
| *FFPs* | 2.60 | 5.23 | 2.63 | **0.0452*** |
| *Platelets* | 0.38 | 3.36 | 2.99 | **0.0461*** |
| *Cryo* | 0.47 | 0.41 | -0.06 | 0.4302 |
| *Crystalloids (L)* | 1.60 | 3.12 | 1.53 | **0.0055*** |
|  |  |  |  |  |
| **Next 20 hours** |  |  |  |  |
| *PRBCS* | 8.89 | 0.55 | -8.34 | **< 0.0001*** |
| *FFPs* | 8.00 | 0.45 | -7.55 | **< 0.0001*** |
| *Platelets* | 1.04 | 0.09 | -0.95 | **0.0005*** |
| *Cryo* | 0.36 | 0.23 | -0.13 | 0.3389 |
| *Crystalloids (L)* | 6.56 | 7.57 | 1.00 | 0.2255 |
|  |  |  |  |  |
| **24 hours** |  |  |  |  |
| *PRBCS* | 12.00 | 6.09 | -5.91 | **0.0035*** |
| *FFPs* | 10.60 | 5.68 | -4.92 | **0.0140*** |
| *Platelets* | 1.42 | 3.45 | 2.03 | 0.1243 |
| *Cryo* | 0.82 | 0.64 | -0.19 | 0.3362 |
| *Crystalloids (L)* | 8.16 | 10.69 | 2.53 | 0.0769 |
|  |  |  |  |  |

Comparison of the 67 pre and postTEG patients who underwent operative intervention
